# Is the COVID-19 epidemic affecting the body mass of Chinese teenagers? – A longitudinal follow-up study

**DOI:** 10.3389/fpubh.2023.1269076

**Published:** 2023-11-23

**Authors:** Guoyang Qin, Hui Li, Yong Qin, Bin Liu

**Affiliations:** ^1^College of Physical Education, Shandong Normal University, Jinan, China; ^2^Physical Education Teaching and Research Group, Shandong Water Conservancy Vocational College, Rizhao, China; ^3^Physical Education Teaching and Research Office, Lixia District Bureau of Education and Sports of Jinan City, Jinan, China; ^4^Physical Education Teaching and Research Group, Jinan Licheng No.2 High School, Jinan, China

**Keywords:** body mass index, COVID-19, physical health, adolescents, follow-up study

## Abstract

**Background:**

After the spread and outbreak of COVID-19 worldwide, the learning, lifestyle, and health level of young individuals have been immensely impacted. With regard to the existing studies, the development trend of adolescents’ body shape in the late COVID-19 period is not sufficiently analyzed, and relevant targeted investigation is lacking. This study aimed to explore the body mass index (BMI) changes of 6–14 years-old adolescents before and after the COVID-19 epidemic, and to provide a reference for promoting the continuous enhancement of adolescent health.

**Methods:**

The BMI and related data pertaining to 93,046 individuals from 2019 to 2022 were collected by cluster sampling, and changes in the BMI *Z* score and detection rate of overweight and obese adolescents before and after the epidemic were analyzed. Furthermore, the trend of obesity rates among adolescents in Jinan from 2019 to 2022 was analyzed using a logistic regression analysis model.

**Results:**

The one-way ANOVA models indicated that the BMI *Z* score of 6–14 years-old adolescents in 2020 significantly increased compared to 2019 (*p* < 0.01), and decreased in 2021 and 2022; in 2020, the obesity rate of adolescents exhibited a significant increase; however, the rate decreased after being controlled in 2021 and 2022. The normal-body size proportion continued to rise (*p* < 0.01), and adolescents of different age groups and genders exhibited similar development trends; the results of the logistic regression analysis indicate that there was a significant increase in obesity rates in 2020, adolescents of different age groups and genders exhibited similar development trends (*p* < 0.05).

**Conclusion:**

This study demonstrated that the COVID-19 epidemic impacts the BMI and obesity detection rate of adolescents. Adolescents from different age groups and genders exhibited similar development trends.

## Highlights

- In 2020, there was a significant increase in BMI *Z* scores and obesity rates among adolescents; however, they were controlled and decreased in 2021 and 2022.
- Adolescents from different age groups and genders exhibited similar development trends.

## Introduction

During the end of 2019, the corona virus (COVID-19) emerged and spread in China. Due to the spread of the epidemic, researchers are anticipating a major worldwide health event in 2020 (1). COVID-19 is characterized by uncertainty of variation, complexity of prevention and control, and rapid spread (2), which crucially threaten the physical and mental health of the individuals and the normal development of the social economy (3). Based on the growing problem of overweight adolescents and obesity among adolescents, more than 50% of adult residents in China are overweight and obese; similarly, nearly 20% of adolescents are overweight and obese (4). Specifically, to control the spread of COVID-19, according to the government’s recommendations, Chinese residents choose home isolation and protection, and schools opt for online teaching. The reduction of outdoor activities and the increased sitting time among teenagers during home isolation may lead to adverse health effects, such as weight gain and declined physical and mental health levels (5). Therefore, to maintain optimal health, weight monitoring is crucial for adolescents.

During the adolescence stage, which is a critical period during which children transform into adults, their physical and mental development matures (6). The self-consciousness of adolescents increases with age, their emotional stability becomes poor, and their physical and mental health becomes vulnerable to the spread of the COVID-19 (7). COVID-19 has exerted an immense impact on the body shape and dietary habits of adolescents. Studies have indicated that the epidemic significantly impacts the detection rate of overweight and obese adolescents, and the poor dietary proportion (8, 9). Some research has observed that during the COVID-19 epidemic, weight, carbohydrate intake and drinking among adolescents have shown an upward trend (10–12). The body mass index (BMI), a universally accepted health marker, is widely utilized to measure malnutrition, determine whether individuals are overweight, and measure obesity (13–15). However, current research on the development trend of Chinese adolescents’ body shape before and after the COVID-19 epidemic is unclear, and a lack of relevant targeted investigation is apparent.

This study aimed to analyze changes in the physical shape and health level of Chinese adolescents before and after the outbreak of COVID-19. We hypothesized that the COVID-19 epidemic significantly affects the health level of adolescents’ body shape in China.

## Materials and methods

### Subjects

The data were collected from a national survey on physical fitness conducted among primary and junior schools between 2019 and 2022 in Jinan, Shandong province, China. 6–14 years-old children completed the measurement and collection of body shape data, and judged various categories such as low weight, normal, overweight and obesity according to the Chinese national standards “Screening for Malnutrition in School Age Children and Adolescents (WS/T 456-2014)” and “Screening for Overweight and Obesity in School Age Children and Adolescents (WS/T 586-2018).” For all participants, both participants and their parents (or guardians) provided their informed consent. The study protocol complied with the Declaration of Helsinki and was approved by the Ethics Committee of Shandong Normal University (2021036).

### Procedures

Based on the technical specifications including the “National Student Physical Health Standard,” we conducted anthropometric measurements. We adopted intelligent physical health monitoring equipment, such as a height tester (GMCS-SGJ3) and weight tester (GMCS-RCS3), and we utilized non-contact measurement using the infrared multi-point sensor array, which automatically recorded the students’ data and uploaded these values to the system for storage.

#### Height and weight

In the process of measuring the height and weight indicators of teenagers, students are required to take off their shoes and hats, and to stand on the bottom plate of the measuring instrument with their backs facing the pillar, their torso naturally straight, and their eyes facing forward. The upper limbs naturally droop, and the legs straighten. The students are required to keep the heels together, their toes approximately 60° apart, and their heels, sacrum, and scapula in contact with the column; thus, they can maintain the standing posture of “three points and one line,” with the unit of cm and kg. The accuracy is set at 1 decimal place (16). The height and weight data of teenagers have been measured only once, and calculate the incidence rate of overweight and obesity among adolescents.

### BMI calculation

The BMI was calculated using the following formula: BMI = weight (kg)/height (m)^2^. Students were divided into four categories based on their BMI values according to the World Health Organization (WHO) criteria, namely <18.5 kg/m^2^, 18.5–23.9 kg/m^2^, 24–27.9 kg/m^2^, and ≥28 kg/m^2^, which represent underweight, average weight, overweight, and obese individuals, respectively (17). According to the WHO growth curve for children and adolescents of different ages and genders (18), the BMI *Z* score for each participant was calculated using the following formula: BMI *Z* score = (measurement value − median of the same age and gender reference standard)/standard deviation of the reference standard (19). A >1 BMI *Z* score indicates that adolescents are overweight; a >2 BMI *Z* score indicates obesity in adolescents; and a <−2 BMI *Z* score indicates weight loss in adolescents (20).

## Calculation

### Statistical analysis

Experimental data were processed using the IBM SPSS statistical software (version 26.0, Chicago, IL, United States). All data were presented as “mean ± standard deviation” (M ± SD). An independent sample *t*-test, *z*-test or one-way analysis of variance (ANOVA) was conducted to compare the mean differences among groups. The logistic regression analysis model was utilized to determine the trends in the body shape compliance rate throughout the study duration, and the significance level was set at *p* < 0.05 for all tests.

## Results

### Descriptive analysis

This study included a total of 93,046 adolescents aged 6–14 in Jinan City from 2019 to 2022. 21,966, 23,049, 23,607, and 24,424 individuals were collected from 2019 to 2022, including 48,790 males (52.44%) and 44,256 females (47.56%). In the data samples collected from 2019 to 2022, there was no statistically significant difference in age between adolescent boys and girls (*p* > 0.05), and boys exhibited higher height, weight, BMI, and BMI *Z* scores than girls (*p* < 0.01). (height in 2019, 2020, 2021, 2022)[boys: 150.60 ± 17.98 cm, 151.09 ± 17.82 cm, 151.51 ± 18.25 cm, 150.54 ± 18.52 cm; girls: 147.57 ± 15.45 cm, 148.20 ± 15.66 cm, 148.35 ± 15.86 cm, 147.79 ± 16.23 cm]; (weight in 2019, 2020, 2021, 2022)[boys: 48.27 ± 19.51 kg, 49.01 ± 20.14 kg, 48.67 ± 20.14 kg, 47.62 ± 19.69 kg; girls: 43.20 ± 15.50 kg, 43.52 ± 16.04 kg, 43.44 ± 16.13 kg, 42.81 ± 15.97 kg]; (BMI *Z* scores in 2019, 2020, 2021, 2022)[boys: 0.78 ± 0.98, 0.80 ± 0.99, 0.74 ± 0.95, 0.73 ± 1.05; girls: 0.36 ± 0.69, 0.39 ± 0.70, 0.35 ± 0.69, 0.34 ± 0.76].

In 2020, the BMI *Z* score of 6–14 years-old adolescents in Jinan City significantly increased compared to 2019 and was higher than the maximum values in 2019, 2021, and 2022 (2019: 0.56 ± 0.88; 2020: 0.63 ± 0.89; 2021: 0.56 ± 0.86; 2022: 0.54 ± 0.94; *F* = 18.354, *p* < 0.01). The BMI *Z* scores of 6–14 years-old adolescents in all age groups exhibited a similar development trend, with a significant increase in BMI *Z* scores in 2020 and a decrease in 2021 and 2022.

By comparing the low weight, normal, overweight, and obesity detection rates among adolescents in Jinan from 2019 to 2022, it is observed that with the outbreak of COVID-19, the obesity rate of adolescents in all groups increased to varying degrees in 2020, and the proportion of normal body shape declined. With the enhancement of epidemic prevention and control as well as the adaptability of adolescents, the obesity rate has been controlled and decreased in 2021 and 2022, and the proportion of normal body size has been continuously increasing (2019:62.32%; 2020: 61.01%; 2021: 62.73%; 2022: 63.57%, trend *X*^2^ = 44.359, *p* < 0.01, Cramer’s *V* = 0.018). Teenagers of different academic stages and genders exhibit similar development trends.

### Regressive analysis

After adjusting for age, grade, and gender factors, a logistic regression analysis was conducted to investigate the changes in overweight and obesity overweight and obesity rates among boys, girls, primary school students, middle school students, and total students. The logistic regression analysis indicated that the changing trend of the adolescent obesity rate in Jinan City from 2019 to 2022 was analyzed. Using a regression analysis, it was observed that the detection rate of adolescent obesity in 2020 exhibited a significant increase compared to 2019, and decreased in 2021 and 2022, with a statistically significant change (*p* < 0.05). Moreover, the study observed a similar trend of change between boys and girls (boys: OR = 1.087, 95% CI = 1.031–1.146, *p* < 0.05; girls: OR = 0.957, 95% CI = 0.903–1.015, *p* < 0.05), as well as between primary and middle school stages (primary and middle school stage: OR = 1.103, 95% CI = 1.039–1.17, *p* < 0.05; middle-school stage: OR = 1.103, 95% CI = 1.039–1.17, *p* < 0.05). The obesity detection rate significantly increased in 2020 and decreased in 2021 and 2022.

## Discussion

The study conducted the first longitudinal survey on the changes and trends in indicators (e.g., BMI and obesity rate) among Chinese adolescents before and after the COVID-19 epidemic. This study demonstrated that the BMI *Z* score and obesity rate of adolescents aged 6–14 in 2020 have significantly increased compared to 2019, and have been controlled to decrease in 2021 and 2022. The proportion of normal body size continues to increase in 2021 and 2022, and adolescents of different academic stages and genders exhibit similar development trends. The results of this study indicates that COVID-19 exerts a negative impact on the physical shape and health of young individuals. With the enhanced epidemic control and adaptability, young individuals exhibit a more healthy physical shape.

The results of this study indicates that the weight and obesity rate of adolescents increased after the COVID-19 epidemic, and the preceding conclusion is consistent with the relevant research results. Wen (21) and Jia’s research (22) recruited 10,082 and 19,066 adolescents from China, respectively, and the comparison indicated that the obesity rate of adolescents increased from 10.5 to 12.6% before and after the epidemic. Another survey indicated that the obesity rate increased from 10.47 to 12.28%. Researchers from countries including Germany (23) and Greece (24) noted that during the COVID-19 epidemic, the weight of adolescents exhibited an increasing trend. According to the results, the weight and obesity rate of adolescents are exhibiting an increasing trend, similar to that described in a previous study. In the late COVID-19 period, with the enhancement of adolescents’ adaptability and prevention and control level, the BMI *Z* score and obesity rate of 6-14-year-old individuals declined, and the proportion of normal sized adolescents continued to rise.

The regression analysis results indicate that the detection rate of adolescent obesity in 2020 exhibited a significant increase compared to 2019, and decreased in 2021 and 2022. Moreover, the study observed a similar trend of change between males and females, as well as between the primary and middle school stages. The aforementioned conclusion is consistent with the relevant research results. Huang’s study (25) revealed that in the COVID-19 context, the weight gain risk increased with the increase in transmission time of COVID-19. During the COVID-19 epidemic, the significantly increased obesity rate of adolescents was related to the home isolation management mode. Due to the reduction of outdoor activities and the increase of sedentary time among adolescents, obesity became a more critical research topic.

However, this study exhibits some limitations. First, the study, which collected and analyzed body shape data for only primary and middle school stages, did not comprehensively consider the entire growth cycle of adolescents. Therefore, the development status of adolescents in each stage should be comprehensively considered. Second, because >95% study participants were from Jinan City, Shandong Province, the sample did not comprehensively represent the adolescents’ population in China. Studies with larger sample sizes and comprising participants from different provinces, as well as other cohorts (e.g., age), are warranted to examine and confirm the observations recorded herein (see Tables 1–4).

**Table 1:** Mean and standard deviation of age, height, weight, BMI, BMI *Z* score of Adolescents in Jinan City from the years 2019 to 2022.

Years	Gender	Statistical value	Age	Height (cm)	Weight (kg)	BMI (kg m^−2^)	BMI *Z* score
2019	Male		10.22 ± 2.68	150.60 ± 17.98^a^	48.27 ± 19.51^b^	20.41 ± 4.77^c^	0.78 ± 0.98^d^
	Female		10.24 ± 2.65	147.57 ± 15.45^a^	43.20 ± 15.50^b^	19.19 ± 4.11^c^	0.36 ± 0.69^d^
		*t*	−0.554	13.334	21.161	20.142	35.569
		*P*	0.579	<0.01	<0.01	<0.01	<0.01
2020	Male		10.24 ± 2.66	151.09 ± 17.82^a^	49.01 ± 20.14^b^	20.57 ± 4.97^c^	0.80 ± 0.99^d^
	Female		10.27 ± 2.66	148.20 ± 15.66^a^	43.52 ± 16.04^b^	19.23 ± 4.20^c^	0.39 ± 0.70^d^
		*t*	−0.822	13.026	22.760	23.759	34.997
		*P*	0.411	<0.01	<0.01	<0.01	<0.01
2021	Male		10.21 ± 2.70	151.51 ± 18.25^a^	48.67 ± 20.14^b^	20.28 ± 4.80^c^	0.74 ± 0.95^d^
	Female		10.26 ± 2.68	148.35 ± 15.86^a^	43.44 ± 16.13^b^	19.05 ± 4.21^c^	0.35 ± 0.69^d^
		*t*	−1.608	14.138	21.883	20.969	36.288
		*P*	0.108	<0.01	<0.01	<0.01	<0.01
2022	Male		10.07 ± 2.69	150.54 ± 18.52^a^	47.62 ± 19.69^b^	20.12 ± 4.82^c^	0.73 ± 1.05^d^
	Female		10.12 ± 2.78	147.79 ± 16.23^a^	42.81 ± 15.97^b^	18.91 ± 4.25^c^	0.34 ± 0.76^d^
		*t*	−1.424	12.321	20.846	20.761	33.952
		*P*	0.154	<0.01	<0.01	<0.01	<0.01

a Significant statistical difference between boys and girls in height, *p* < 0.05.

b Significant statistical difference between boys and girls in weight, *p* < 0.05.

c Significant statistical difference between boys and girls in BMI, *p* < 0.05.

d Significant statistical difference between boys and girls in BMI *Z* score, *p* < 0.05.

**Table 2:** Mean and standard deviation of BMI *Z* score of Adolescents in Jinan City from the years 2019 to 2022.

Age	2019		2020		2021		2022		*F*	*P*
	*n*	M ± SD	*n*	M ± SD	*n*	M ± SD	*n*	M ± SD		
6	2,420	0.28 ± 0.87^a^	2,472	0.41 ± 0.91^a^	2,642	0.32 ± 0.88^a^	3,002	0.36 ± 1.39^a^	7.236	<0.01
7	2,402	0.49 ± 0.95^b^	2,422	0.58 ± 0.99^b^	2,483	0.45 ± 0.92^b^	2,652	0.46 ± 0.94^b^	9.089	<0.01
8	2,223	0.63 ± 1.01^c^	2,407	0.65 ± 1.00^c^	2,407	0.58 ± 0.99^c^	2,476	0.56 ± 0.93^c^	4.499	<0.01
9	2,106	0.67 ± 0.95^d^	2,213	0.73 ± 0.99^d^	2,393	0.64 ± 0.92^d^	2,441	0.64 ± 0.91^d^	5.184	<0.01
10	1,797	0.69 ± 0.89^e^	2,116	0.73 ± 0.94^e^	2,191	0.68 ± 0.96^e^	2,406	0.61 ± 0.89^e^	4.399	<0.01
11	2,000	0.71 ± 0.89^f^	1,788	0.78 ± 0.90^f^	2,058	0.71 ± 0.86^f^	2,176	0.68 ± 0.85^f^	4.209	<0.01
12	3,385	0.61 ± 0.82^g^	3,439	0.68 ± 0.83^g^	2,832	0.64 ± 0.79^g^	3,201	0.61 ± 0.81^g^	4.660	<0.01
13	2,815	0.53 ± 0.75^h^	3,384	0.59 ± 0.77^h^	3,347	0.57 ± 0.77^h^	2,735	0.51 ± 0.72^h^	4.714	<0.01
14	2,818	0.47 ± 0.68^i^	2,808	0.55 ± 0.75^i^	3,254	0.49 ± 0.71^i^	3,335	0.45 ± 0.72^i^	11.213	<0.01
Total	21,966	0.56 ± 0.88^j^	23,049	0.63 ± 0.89^j^	23,607	0.56 ± 0.86^j^	24,424	0.54 ± 0.94^j^	18.354	<0.01

a Significant statistical difference in 2019, 2020, 2021, and 2022 for age 6, *p* < 0.05.

b Significant statistical difference in 2019, 2020, 2021, and 2022 for age 7, *p* < 0.05.

c Significant statistical difference in 2019, 2020, 2021, and 2022 for age 8, *p* < 0.05.

d Significant statistical difference in 2019, 2020, 2021, and 2022 for age 9, *p* < 0.05.

e Significant statistical difference in 2019, 2020, 2021, and 2022 for age 10, *p* < 0.05.

f Significant statistical difference in 2019, 2020, 2021, and 2022 for age 11, *p* < 0.05.

g Significant statistical difference in 2019, 2020, 2021, and 2022 for age 12, *p* < 0.05.

h Significant statistical difference in 2019, 2020, 2021, and 2022 for age 13, *p* < 0.05.

i Significant statistical difference in 2019, 2020, 2021, and 2022 for age 14, *p* < 0.05.

j Significant statistical difference in 2019, 2020, 2021, and 2022 for total, *p* < 0.05.

**Table 3:** Detection rates of low weight, normal, overweight, and obesity among 6–14 years-old adolescents in Jinan City from 2019 to 2022.

Years	Low weight (numbers)	Normal (numbers)	Overweight (numbers)	Obesity (numbers)	Trend *X*^2^	*P*	Cramer’s *V*
Male					16.363	<0.01	0.021
2019	3.13% (363)	58.73% (6,820)	16.49% (1,915)	21.65% (2,515)^d^			
2020	3.59% (433)	56.27% (6,772)	16.53% (1,989)	23.61% (2,841)			
2021	3.50% (433)^a^	58.54% (7,242)	16.83% (2,082)	21.13% (2,614)			
2022	3.46% (442)	59.28% (7,570)^b^	17.47% (2,231)^c^	19.79% (2,528)			
Female					29.339	<0.01	0.018
2019	2.98% (309)	66.35% (6,869)	15.84% (1,640)^c^	14.83% (1,535)^d^			
2020	4.04% (445)	64.37% (7,090)	16.11% (1,774)	15.48% (1,705)			
2021	3.71% (417)	67.34% (7,566)	14.89% (1,673)	14.06% (1,580)			
2022	3.89% (453)^a^	68.28% (7,957)^b^	14.86% (1,732)	12.97% (1,511)			
Primary school					27.062	<0.01	0.020
2019	3.44% (445)	64.46% (8,346)	15.29% (1,980)	16.81% (2,177)^d^			
2020	4.90% (657)	63.67% (8,543)	15.34% (2,058)	16.09% (2,160)			
2021	4.54% (644)^a^	65.79% (9,325)^b^	14.63% (2,074)	15.04% (2,131)			
2022	4.30% (651)	65.44% (9,916)	15.31% (2,321)^c^	14.95% (2,265)			
Middle school					9.542	<0.01	0.025
2019	2.52% (227)	59.24% (5,343)	17.47% (1,575)	20.77% (1,873)			
2020	2.29% (221)	57.3% (5,519)	16.67% (1,605)	23.74% (2,286)			
2021	2.18% (206)	58.13% (5,483)	17.82% (1,681)^c^	21.87% (2,063)^d^			
2022	2.63% (244)^a^	60.53% (5,611)^b^	17.71% (1,642)	19.13% (1,774)			
Total					44.359	<0.01	0.018
2019	3.06% (672)	62.32% (13,689)	16.18% (3,555)	18.44% (4,050)^d^			
2020	3.81% (878)	61.01% (14,062)	15.89% (3,663)	19.29% (4,446)			
2021	3.60% (850)	62.73% (14,808)	15.90% (3,755)	17.77% (4,194)			
2022	3.66% (895)^a^	63.57% (15,527)^b^	16.23% (3,963)^c^	16.54% (4,039)			

a Comparison of the highest low weight rates in 2019, 2021, and 2022 with 2020, *p* < 0.05.

b Comparison of the highest normal rates in 2019, 2021, and 2022 with 2020, *p* < 0.05.

c Comparison of the highest overweight rates in 2019, 2021, and 2022 with 2020, *p* < 0.05.

d Comparison of the highest obesity rates in 2019, 2021, and 2022 with 2020, *p* < 0.05.

**Table 4:** Logistic regression analysis of low weight, normal, overweight, and obesity detection among 6–14 years-old adolescents in Jinan City from 2019 to 2022.

Years	OR(95%CI)				
	Male	Female	Primary school	Middle school	Total
2019	1.000^a^	1.000^b^	1.000^c^	1.000^d^	1.000^e^
2020	1.087 (1.031–1.146)^a^	0.957 (0.903–1.015)^b^	0.972 (0.923–1.025)^c^	1.103 (1.039–1.17)^d^	1.027 (0.988–1.068)^e^
2021	0.990 (0.939–1.043)^a^	0.917 (0.865–0.972)^b^	0.887 (0.842–0.935)^c^	1.069 (1.007–1.134)^d^	0.956 (0.919–0.994)^e^
2022	0.974 (0.925–1.027)^a^	0.873 (0.824–0.926)^b^	0.917 (0.871–0.965)^c^	0.948 (0.893–1.007)^d^	0.927 (0.892–0.964)^e^
*P*	<0.05	<0.05	<0.05	<0.05	<0.05

a Significant statistical difference in 2019, 2020, 2021, and 2022 for male, *p* < 0.05.

b Significant statistical difference in 2019, 2020, 2021, and 2022 for female, *p* < 0.05.

c Significant statistical difference in 2019, 2020, 2021, and 2022 for primary school, *p* < 0.05.

d Significant statistical difference in 2019, 2020, 2021, and 2022 for middle school, *p* < 0.05.

e Significant statistical difference in 2019, 2020, 2021, and 2022 for total, *p* < 0.05.

## Conclusion

In 2020, the BMI *Z* score and obesity rate of 6–14 years-old adolescents exhibited a significant increase compared to 2019, and were controlled to decrease in 2021 and 2022. The normal body size proportion continued to rise, and adolescents of different age groups and genders exhibited similar development trends.

## Data availability statement

The original contributions presented in the study are included in the article/supplementary material, further inquiries can be directed to the corresponding author.

## Ethics statement

The studies involving humans were approved by Ethics Committee of Shandong Normal University (2021036). The studies were conducted in accordance with the local legislation and institutional requirements. Written informed consent for participation in this study was provided by the participants’ legal guardians/next of kin.

## Author contributions

GQ: Writing – original draft. HL: Data curation, Writing – review & editing. YQ: Data curation, Writing – review & editing. BL: Data curation, Writing – review & editing.
